# Detecting structural variations with precise breakpoints using low-depth WGS data from a single oxford nanopore MinION flowcell

**DOI:** 10.1038/s41598-022-08576-4

**Published:** 2022-03-16

**Authors:** Henry C. M. Leung, Huijing Yu, Yifan Zhang, Wing Sze Leung, Ivan F. M. Lo, Ho Ming Luk, Wai-Chun Law, Ka Kui Ma, Chak Lim Wong, Yat Sing Wong, Ruibang Luo, Tak-Wah Lam

**Affiliations:** 1 Department of Computer Science, The University of Hong Kong, Pok Fu Lam, Hong Kong; 2 Clinical Genetic Service, Department of Health, Kowloon Bay, Hong Kong; 3 L3 Bioinformatics Limited, Sai Ying Pun, Hong Kong

**Keywords:** Computational biology and bioinformatics, Genetics

## Abstract

Structural variation (SV) is a major cause of genetic disorders. In this paper, we show that low-depth (specifically, 4×) whole-genome sequencing using a single Oxford Nanopore MinION flow cell suffices to support sensitive detection of SV, particularly pathogenic SV for supporting clinical diagnosis. When using 4× ONT WGS data, existing SV calling software often fails to detect pathogenic SV, especially in the form of long deletion, terminal deletion, duplication, and unbalanced translocation. Our new SV calling software SENSV can achieve high sensitivity for all types of SV and a breakpoint precision typically ± 100 bp; both features are important for clinical concerns. The improvement achieved by SENSV stems from several new algorithms. We evaluated SENSV and other software using both real and simulated data. The former was based on 24 patient samples, each diagnosed with a genetic disorder. SENSV found the pathogenic SV in 22 out of 24 cases (all heterozygous, size from hundreds of kbp to a few Mbp), reporting breakpoints within 100 bp of the true answers. On the other hand, no existing software can detect the pathogenic SV in more than 10 out of 24 cases, even when the breakpoint requirement is relaxed to ± 2000 bp.

## Introduction

Structural variation (SV) refers to the changing of copy number, orientation or position of DNA segment; it occurs in the form of deletion, duplication, inversion, and unbalanced/balanced translocation^1^. Genetic disorders caused by pathogenic SV are often heterozygous in nature^2^. Precise detection of SV breakpoint^3^ is important in clinical diagnosis to determine the exact genes affected by an SV and its functional impact. Improving breakpoint precision to ± 100 bp also allows cost-effective PCR verification of the target pathogenic SV in patients’ or relatives’ samples. However, traditional SV detection technologies, such as array CGH, karyotyping, and Next Generation Sequencing (NGS)^4–6^, have low breakpoint resolution and detection sensitivity^7,8^. Latest developments exploit the possibility of using the error-prone third-generation long-read sequencing (3GS), including Oxford Nanopore Technologies (ONT) as a more sensitive detection alternative^9^. Long-reads are capable of capturing a lot more information surrounding the SV breakpoint compared with the NGS short-reads, thus improving the detection sensitivity and precision, especially in those large repetitive regions^6^. With only a few hundred US dollars, current ONT MinION sequencing using a single flow cell can generate approximately 4× human WGS data (determined by in-house experiments). Detection of SV with ONT long-reads, especially for low-coverage data, remains challenging. Intuitively, an SV will give rise to a split-read alignment at its breakpoint in the sense that (1) a read is aligned with a big gap, or (2) the prefix and suffix of the read aligned separately to different parts of the genome. For the former case, existing alignment software often relies on reducing the gap penalty in aligning a read to favor finding a big gap^10,11^. However, a split-read alignment might still be missed if the breakpoint occurs towards the end of a read, or if the SV is too large (i.e., pathogenic SV of hundreds of kbp) for the gap penalty to be worth considering. For the latter case, in order to align the prefix and suffix of a read separately, it requires the read to have a sufficiently long prefix and suffix on each side of the SV. Using low-depth data, there may not be any such reads. On the other hand, as the error rate of 3GS data is higher than NGS, a false positive split-read alignment may be introduced. When using low-depth data, there might not be sufficient correctly aligned reads in the same region to confirm a false positive, thus causing a large number of false-positive SVs. Due to the above limitation, existing tools, including Sniffles^10^, SVIM^12^, cuteSV^13^, require high-depth (i.e., 30 ×) data for SV detection which often exceed the limit of clinical application cost^14^ compared with the traditional methods. NanoVar^14^ was the first attempt to demonstrate the possibility of using low-depth ONT WGS data for SV detection, although the tool is trained with simulated data and performance on real patient data is not guaranteed.

In this study, we consider the definition of SV with the size of at least one thousand base pairs^2,15,16^ and, in particular pay attention to pathogenic SV which often involves over a hundred thousand base pairs^2^. Using 4× ONT data, existing software can still detect an acceptable number of SVs when benchmarked against a normal reference sample like HG002, but they often fail to detect pathogenic SVs when evaluated using patient samples. Notice that almost all the confirmed SVs in HG002 are deletions with less than 100 kbp, yet known pathogenic SVs are much longer, often more than 100 kbp (over 95% of confirmed pathogenic SVs in dbVar have more than 100 kbp in length). Existing software can detect deletion, but the sensitivity drops drastically when the size exceeds 100 kbp; and they have difficulty in detecting duplication, terminal deletion and unbalanced translocation^17^.

To overcome the difficulty in detecting pathogenic SV using 4× ONT data, we devised a new software tool called SENSV to detect SV with better sensitivity for all sizes (including over 100 kbp) and for all types of SV. SENSV achieves a breakpoint precision ± 100 bp. To identify genome intervals with potential copy number variation (CNV), SENSV compares the sequencing depth of each 10 K genome interval against that of 24 sequenced 4× ONT references samples. The related reads are then realigned using an SV-aware aligner to recover most of the poorly aligned or misaligned split-read alignments. SENSV also assesses each detected SV by realigning the related reads to an altered reference sequence with the conjectured SV and applies filter metrics including alignment score, sequencing depth and allele frequency to remove false positives further. In this study, we evaluated SENSV together with the state-of-the-art ONT SV callers using real patient samples. SENSV outperformed others with breakpoint detected as precise as ± 100 bp, especially for handling difficult cases including long deletion (i.e., over 100 kbp), duplication, terminal deletion and unbalanced translocation, by recovering misaligned split reads. In addition, SENSV has demonstrated the best sensitivity among the software in detecting the 532 GIAB-confirmed HG002 deletions (≥ 1 kbp) and seven types of pathogenic variants implanted in two simulated low-depth datasets.

## Results and discussion

We benchmarked SENSV and existing software NanoVar^14^, Sniffles^10^, SVIM^12^ and cuteSV^13^ in detecting heterozygous SVs from low-depth ONT WGS data. We used (1) real data from 24 patients with genetic disorders and experimentally verified SVs; (2) HG002 sequenced by a single MinION flow cell; evaluation is based on a recently published SV set; and (3) two simulated datasets, each with 35 planted SVs of various types.

### Real data from patients with genetic disorders

For the real patient data, DNA was extracted from EDTA blood using Maxwell RSC Whole Blood DNA Kit with Maxwell RSC Instrument (Promega, Spain) and stored at − 80 °C upon sequencing. All DNA samples were prepared using an in-house protocol optimized based on the ONT SQK-LSK109 ligation protocol (GDE_9063_v109_revY_14Aug2019) with a target read-length N50 of approx. 10 kbp before MinION sequencing. Sequencing for each sample was performed using one MinION flowcell for at least 72 h and until the flow cell had less than 50 active pores. Fast5s generated were basecalled using Guppy v.3.1.5 and flip-flop model. The average read length of the sequenced library ranged from 3425.81 to 11,424.75 bp; and N50 range from 6200 to 15,909 bp in this study. The average depth was 4.2× (lowest 2.9×; highest  6.5 ×). The pathogenic SVs of the 24 samples and their rough positions were diagnosed by medical doctors using conventional methods, including array CGH and karyotype analysis. These SVs were all heterozygous. The first ten samples involved a simple deletion or duplication occurring at a normal region of a chromosome; the next six samples were more complicated, involving an unbalanced translocation or terminal deletion occurring near a highly repeated telomere region. Sample 17 to 24 involved an inversion or a balanced translocation. The (true) breakpoints of SVs were determined based on a detailed analysis by a bioinformatician and a medical doctor; ten samples were selected randomly, and the true breakpoints were all confirmed by PCR and/or Sanger sequencing. These true breakpoints were used to evaluate the software tools using SURVIVOR^18^.

The results of the evaluation are shown in Table 1. Samples 1 to 10 (in increasing order of SV length) is concerned with a simple deletion or duplication. SENSV detected 9 out of 10 SVs, reporting breakpoints within 100 bp of the true breakpoints. The best of the other software detected at most five SVs when we relaxed the breakpoint resolution to 2000 bp (marked as Y-). Note that the existing software was not designed for detecting heterozygous SV using WGS data with depth as low as 4× , and they might not find sufficient supporting split-read alignments to detect the SV. Table 1 also suggests that the existing software is not sensitive for detecting large unbalanced SVs (length 638 kbp or more). SENSV can detect large unbalanced SVs using sequencing depth information and recover the precise breakpoint of SV by SV-DP and realignment (samples 4 to 10).

**Table 1:** Comparison of SENSV, NanoVar, Sniffles, SVIM and cuteSV on the ability to detect the pathogenic SV from the 24 patients’ ONT WGS data.

ID	SV length	SV type	Detect the pathogenic SV? (# of predicted SVs of the same type)				
			SENSV	NanoVar	Sniffles	SVIM	cuteSV
**Difficult cases**							
1	146 K	Long deletion	**Y (1513)**	**Y (1278)**	Y- (1027)	**Y (2078)**	**Y (831)**
2	266 K	Long deletion	**Y (1431)**	**Y (1353)**	**Y (1172)**	**Y (2129)**	**Y (945)**
3	638 K	Long deletion	N (1743)	N (1146)	N (732)	N (2207)	N (599)
4	670 K	Long deletion	**Y (1612)**	N (1176)	N (794)	N (2036)	N (658)
5	1.5 M	Long deletion	**Y (3395)**	N (1514)	N (1062)	N (4970)	N (848)
6	1.4 M	Long deletion	**Y (17,209)**	N (3692)	N (1375)	Y- (17,783)	N (834)
7	1.4 M	Duplication	**Y (200)**	N (1779)	N (667)	Y- (1901)	N (2105)
8	2.8 M	Long deletion	**Y (1,792)**	N (1320)	N (1192)	N (2983)	N (1016)
9	5.2 M	Long deletion	**Y (1515)**	N (1294)	N (1014)	N (1982)	N (786)
10	6.6 M	Long deletion	**Y (2868)**	N (1474)	**Y (1362)**	**Y (4534)**	**Y (1090)**
11	342 K	Unbalanced translocation	N (490)	N (7106)	N (1383)	N (1154)	N (1310)
12	1.4 M	Unbalanced translocation	**Y (462)**	N (7802)	N (1432)	N (1179)	N (1326)
13*	5.9 M	Terminal deletion	**Y (3476)**	N (1498)	N (1510)	N (4649)	N (1168)
14	18 M	Unbalanced translocation	**Y (269)**	N (6156)	N (1719)	N (1429)	N (1595)
15	19 M	Terminal deletion	**Y (1413)**	N (1220)	N (968)	N (2031)	N (798)
16	58 M	Unbalanced translocation	**Y (883)**	N (6688)	N (2340)	N (2053)	N (2263)
**Others**							
17	142 K	Inversion	**Y (114)**	Y- (1413)	Y- (1391)	Y- (849)	**Y (1336)**
18*	73 M	Inversion	**Y (118)**	**Y (1333)**	**Y (853)**	**Y (523)**	Y- (903)
19	33 M	Inversion	**Y (192)**	**Y (1258)**	N (190)	**Y (117)**	N (210)
20	N/A	Balanced translocation	**Y (89)**	**Y (5554)**	**Y (1463)**	**Y (1190)**	**Y (1404)**
21*	N/A	Balanced translocation	**Y (861)**	**Y (6898)**	**Y (2594)**	N (2012)	**Y (2518)**
22	N/A	Balanced translocation	**Y (76)**	**Y (5254)**	**Y (1187)**	N (996)	**Y (1084)**
23	N/A	Balanced translocation	**Y (111)**	**Y (5542)**	**Y (1449)**	N (1065)	**Y (1319)**
24	N/A	Balanced translocation	**Y (592)**	**Y (6628)**	**Y (1775)**	**Y (1358)**	**Y (1645)**

Below, “Y” [and “Y-”] mean that a method can detect the pathogenic SV with correct SV type and with breakpoints off by at most 100 bp [and by at most 2000 bp respectively]; and “N” indicates the method unable to detect the SV with breakpoints off by at most 2000 bp. SENSV can detect more SVs, especially for difficult cases, with much fewer false positives. Other software usually detects much more SVs than SENSV but most of them are false positives. The samples ID with asterisk have been basecalled using both Guppy versions (v3.1.5 and v5.0.11).

The best results of a row are in bold.

Samples 11 to 16 contain an unbalanced translocation and terminal deletion near a highly repeated telomere region. They involve a deletion at highly repeated telomere regions on different chromosomes. They are difficult to detect because (1) a read might be aligned to the telomere region of different chromosomes with similar patterns, and (2) the genome reference of the telomere regions of some chromosomes is missing. SENSV can detect five out of six complicated unbalanced SVs (samples 13 to 16) by applying SV-DP to detect the correct alignment, filtering false-negative alignment by depth information and reconstructing the genome reference of each chromosome’s telomere by assembling ONT reads in a 150× standard NA12878 sample. In contrast, the other software tools failed to detect these SVs.

Balanced SVs, which can be detected by a read covering one of the two breakpoints (unbalanced SV can be detected by a read covering exactly one breakpoint only), can usually be detected by existing software (sample 17 to 24). However, more false-positive are predicted for balanced SVs due to 1) chimeric reads introduced by sequencing error and misalignment, and 2) inability to filter false positives by sequencing depth information. In addition, existing software usually predicts an unexpectedly large number of translocations (hundreds to thousands) while a patient with genetic disease is expected to have fewer than 10 translocations^19^. This large number of reported SVs introduces difficulties in distinguishing the disease-causing balanced SVs from the false positives. SENSV, by applying realignment and filtering, limits the number of false positives within a few hundreds. Thus, it can reduce a large effort of distinguishing the disease-causing balanced SVs.

To assess the performance consistency using different versions of basecaller, three of the above samples (i.e. sample 13, 18 and 21) were also basecalled using Guppy v.5.0.11 and SUP model. SENSV is able to detect the same pathogenic SV within 100 bp breakpoint precision in them using the output of different basecallers. We have thereby confirmed that the performance of SENSV is stable under different levels of per base accuracy.

### Real normal DNA data

We also evaluated SENSV by sequencing the normal reference DNA sample HG002, commonly used for evaluating SV calling software. For the standard HG002 sample, pure DNA was ordered from Coriell Institute and stored at -20’C before sequencing as described for real patient samples. Our evaluation is based on a recently published benchmark set^20^ containing 532 confirmed SV with a size of at least 1 kbp. They, except one, are all short deletions with sizes shorter than 100 kbp. The DNA sample was sequenced using a single MinION flow cell, generating 3.7× data. Table 2 shows the sensitivity of SENSV, NanoVar, Sniffles, SVIM and cuteSV on detecting SVs in HG002 with breakpoint precision of 100 bp and 2000 bp, respectively. SENSV detected 390 (73% of 532) GIAB-confirmed SVs with breakpoint precision ± 100 bp; the total number of SVs predicted is 3644. SVIM has the second-best performance; it predicts more SVs (6650 in total) but detects slightly fewer GIAB-confirmed SVs (379; 71% of 532). When breakpoint precision was evaluated using GIAB’s practice of ± 2000 bp, SENSV and SVIM could detect 432 (81% of 532) and 428 (80% of 532) GIAB-confirmed SVs, respectively. Notice that all the 532 confirmed SVs, except one, are short deletion with a size less than 100 kbp.

**Table 2:** The performance of SENSV, NanoVar, Sniffles, SVIM and cuteSV on detecting SVs in HG002 with breakpoint precision of 100 bp and 2000 bp respectively.

	Short deletions (< 100 kbp)		Long deletion (> 100 kbp)	
	**# of confirmed SV detected** ± 100 bp (± 2000 bp)	Sensitivity ± 100 bp (± 2000 bp)	# of confirmed SV detected ± 100 bp (± 2000 bp)	Sensitivity ± 100 bp (± 2000 bp)
**SENSV**	**389 (431)**	**73.26% (81.17%)**	**1 (1)**	**100% (100%)**
**NanoVar**	353 (381)	66.48% (71.75%)	0 (0)	0% (0%)
**Sniffles**	269 (313)	50.66% (58.95%)	0 (0)	0% (0%)
**SVIM**	378 (427)	71.19% (80.41%)	**1 (1)**	**100% (100%)**
**cuteSV**	272 (294)	51.22% (55.38%)	0 (0)	0% (0%)

The benchmark set of HG002 contains 531 confirmed short deletions with size of smaller than 100 kbp and one long deletion with size of larger than 100 kbp. The sensitivity inside the paratheses is measured with the relaxed breakpoint precision of 2000 bp.

The best results of a row are in bold.

For detecting short deletions with a size smaller than 100 kbp, the sensitivity of SENSV is slightly higher than SVIM and much better than NanoVar, Sniffles and cuteSV. As most of the confirmed SVs in HG002 are short deletions with an average length of 4259 bp, they can be detected easily (as the gap for a single split-read alignment is small). Notice that some software tools predicted 2 to 4 times more SVs than SENSV. As the SVs on HG002 are not all detected and confirmed by society, we cannot draw any conclusion on the correctness of the predicted SVs. The number of SV reported by each software tool can be found in Supplementary Material S.1. The precision is in Supplementary Material S.2.

### Simulated patient data

To further evaluate SENSV’s ability to detect long deletions (> 100 kbp), duplications, terminal deletions and unbalanced translocations (i.e., the difficult cases) and evaluate the correctness of the predicted SVs, we generated two simulated “patient genomes”. Each “patient genome” was generated by implanting 35 heterozygous SVs on the human genome reference hg19. The 35 SVs consisted of 5 SV of the following seven types: short deletion, long deletion, duplications, terminal deletions, inversions, balanced translocations and unbalanced translocations. All deletions and duplications were selected randomly from the clinically confirmed SVs in the dbVar^2^ database. As there are fewer than ten confirmed terminal deletion, inversions, and balanced and unbalanced translocations in dbVar, these SV types were simulated using RSVSim^21^. The terminal deletions and unbalanced translocations were randomly selected from either the 5’ end or 3’ end of a chromosome, with one of the breakpoints lie in the telomere region. All SVs were not overlapped. These SVs with known positions were implanted into the reference genome hg19 using RSVSim. NanoSim^22^ was used to generate 12 Gbp (i.e., depth of 4×) of WGS data for each “patient genome”. The maximum read length was set to 20 kbp (similar to our library preparation protocol for real data). Other characteristics of reads, including mismatch rate and length distribution, were trained based on real public data: ONT WGS consortium rel6^23^. In order to demonstrate the ability of SENSV to detect homozygous SV, we have generated simulated datasets consisting of the same implanted SV as above “patient genome”, but implanted with homozygous SV.

We evaluated the performance of SENSV, NanoVar, Sniffles, SVIM and cuteSV on calling the implanted SVs (against the reference genome hg19) with the breakpoints error of 100 bp and 2000 bp, respectively. Table 3 shows the number of SV for each type that the software tools could detect in the two simulated datasets. When the breakpoint resolution was required to be ± 100 bp, SENSV detected 58 out of the 70 SVs (31 out of 40 difficult cases) and predicted a total of 255 SVs. The other four tools detected 33, 33, 32 and 34 SVs (12, 10, 14 and 13 difficult cases and predicted 494, 455, 1140 and 294 SVs); note that existing software tools often miss terminal deletions and unbalanced translocations. Overall speaking, SENSV detected more or equal numbers of implanted SVs for each type. For those SV types that are difficult to be detected, i.e., long deletion, duplication, terminal deletion and unbalanced translocation, SENSV outperformed other software significantly. With the advantages of using depth information, SENSV could recover some of the missing long deletions and duplications whose split-reads were not found by the aligners used by other software tools. As a result, it could detect 7 to 11 more deletions and duplications than other software tools. For terminal deletion and unbalanced translocation with one breakpoint lies in the telomere region, existing software tools usually cannot distinguish reads sequenced from different telomere regions of different chromosomes or different positions in the telomere regions. SENSV could distinguish them based on a more accurate alignment of reads in the telomere region or the nearby sub-telomere region. Thus, SENSV can detect 14 (out of 20) terminal deletion and unbalanced translocation, while the second-best software tool, cuteSV, could detect six only. For the other cases that can be detected relatively easier, i.e., short deletion, inversion and balanced translocation, SENSV shared similar performance with other software tools. In addition, the number of SV detected by SENSV using the “homozygous SV” simulation dataset has increased from 58 to 67, indicating that SENSV is able to detect homozygous SVs as well.

**Table 3:** The number of SVs detected with a breakpoint precision of 100 bp by the software grouped by SV types in two simulated datasets.

	# of Detected SVs for the 10 implanted SVs				
	SENSV	NanoVar	Sniffles	SVIM	cuteSV
**Difficult cases**					
Long deletion (> 100 kbp)	**9**	6	3 (4)	5 (6)	3
Duplication	**8**	4	4	5	4
Terminal deletion	**7**	0	0	2	3
Unbalanced translocation	**7**	2	3	2	3
**Others**					
Short deletion (< 100 kbp)	**10**	4	7	**10**	6
Inversion	**9**	**9**	**9**	5	8
Balanced translocation	**8**	**8**	7	3	7

The number in the parentheses is the number of SVs detected with a breakpoint precision of 2000 bp. Each type has total 10 SVs implanted in two simulated datasets.

The best results of a row are in bold.

We have also evaluated those SVs that SENSV cannot detect. Almost all of them were due to no reads being sequenced covering the breakpoints of the planted SVs. There is one exception: one split-read can cover the breakpoint of a balanced translocation but is missed by SENSV. Although SENSV can find the split-read alignment, it did not report the translocation because the alignment score is not good enough. The details of the performance for each SV in each simulated dataset can be found in Supplementary Section S.3. The corresponding recall and precision are in Supplementary Material S.2. We also evaluated the CPU usage of SENSV. The numbers are in Supplementary Material S.4.

## Method

**Figure 1:**
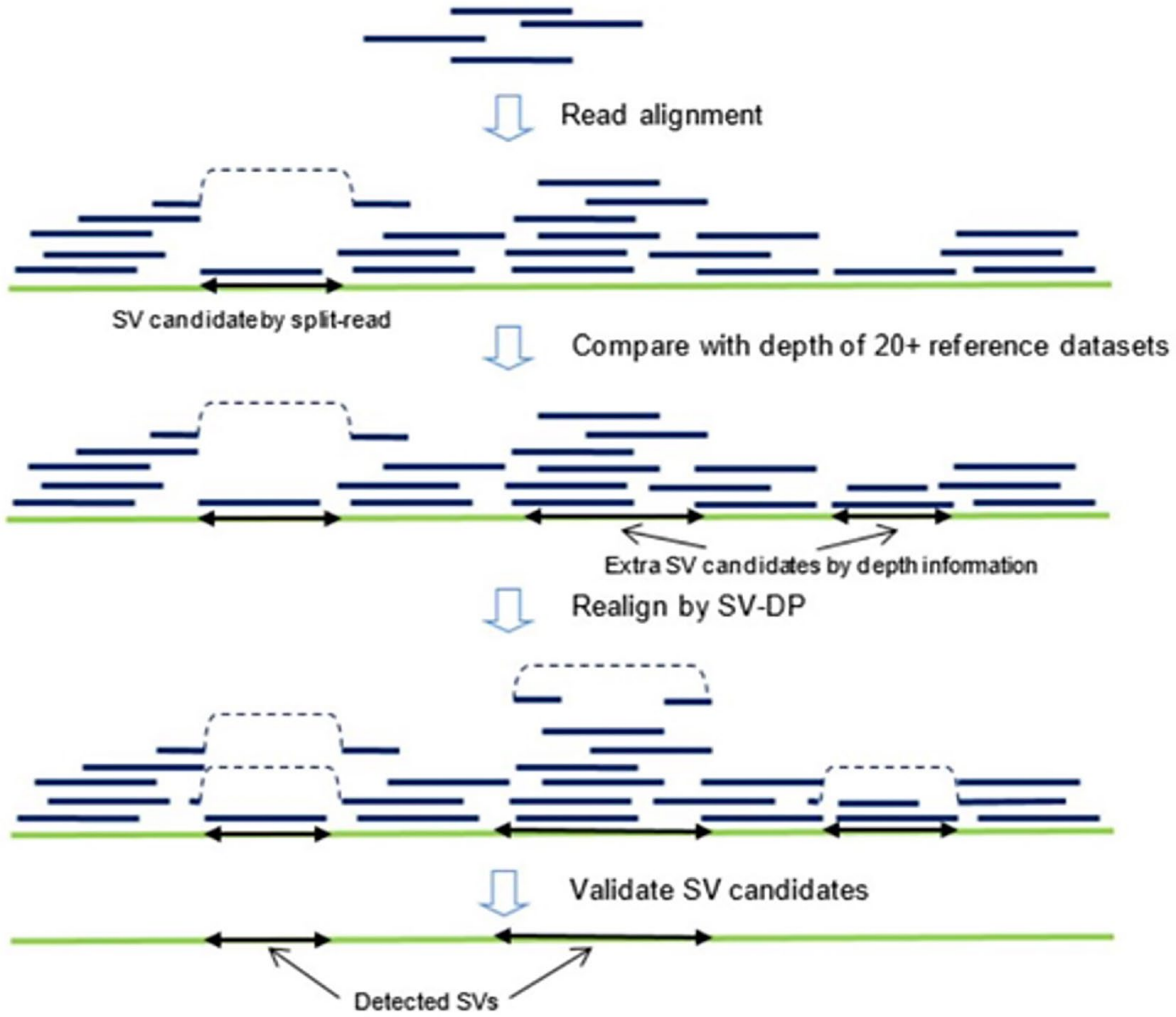
The workflow of SENSV.

SENSV is a computational method for detecting SVs using low-depth ONT WGS data. Figure 1 shows the workflow of SENSV, which has four major steps: (1) SENSV obtains SV candidates from the alignment information of raw reads to the human reference genome. (2) It compares the sequencing depth of each DNA region with 24 reference datasets to detect another set of SV candidates. (3) All these SV candidates are refined using SV-DP, an SV-aware dynamic programming algorithm implemented to find precise breakpoints of the SV. (4) The candidates are filtered and refined based on base-level alignment to a modified reference genome. Quality scores of called SVs are also available, making it possible to apply additional filtering.

SENSV makes use of Minimap2^11^ to align long reads to the human reference genome to find initial SV candidates. Minimap2 is very efficient in processing noisy long reads. A split-read alignment, which aligns the prefix and suffix of a read to different positions, is considered an SV candidate. Many SV candidates could be missed, as reads could be misaligned or poorly aligned, SENSV exploits other methods to recover and verify the candidates. Note that existing software often cannot properly align a read near the end of a chromosome, as it might involve a highly repeated telomere region, and the DNA of such a region might be missing from the reference genome. Therefore, we used our assembly of telomere sequence in SENSV. We aligned a 150× ONT WGS data of a standard human sample NA12878 from GIAB^24^ to the reference genome and found the reads aligned to para-telomere sequences of each chromosome. We assembled these reads using miniasm^25^ and used the assembled sequences as an additional reference DNA for read alignment in SENSV.

### SV candidates via sequencing depth

Since fewer reads are sequenced in a deletion region and more reads in a duplication region, an unbalanced SV candidate, in principle, can be detected by analyzing the sequencing depth of each DNA region. However, when the sequencing depth is low (4×), the sequencing bias (i.e., depth variance) in different DNA regions may introduce many false positives and negatives. To solve this problem, SENSV considers only the DNA regions with abnormal depth compared with a reference dataset. The reference dataset is constructed by ONT WGS data from 24 people that shared no common SVs detected by Karyotyping and CGH array. The average depth of these 24 ONT WGS data is 4.3× (Supplementary Fig.  S1), and the depth is normalized to 4× for each sample.

We partition the genome into intervals of 10 kbp. The choice of the window size for binning sequencing depth is determined empirically. With pilot study we used 5 kbp, 10 kbp and 50 kbp on some simulated datasets (5 kbp and 50 kbp both have detected only 39 out of 70 SVs), while 10 kbp window choice led to the highest sensitivity (58 out of 70 SVs). In addition, the analysis speed would reduce dramatically if given a smaller window. While we are working on low-depth samples, having an interval too short might result in insufficient or even no depth, while an interval too long might reduce breakpoint resolution. For each interval, the average depth is compared to that of the corresponding intervals of the 24 reference datasets. The comparison is accomplished by calculating the log-likelihood of two normal distribution models, representing the sequencing depth distribution of the sample with and without SV, respectively. We assume that each interval is independent of each other (given the interval size is sufficiently large), and define the two models as N(*μ*, *σ*^2^) and N(*μ*_SV_, *σ*^2^), where *μ* and *σ*^2^ represent the mean depth and standard deviation of the corresponding intervals in the 24 reference datasets, *μ*_SV_ is set to 0.5*μ* for detecting deletion and 1.5*μ* for detecting duplication (both assuming heterozygous SV). The likelihood ratio test is used to detect whether the depth of the analyzed interval is more likely to follow N(*μ*, *σ*^2^) or N(*μ*_SV_, *σ*^2^), i.e., to be normal or abnormal.

Lastly, nearby abnormal intervals are merged through a greedy algorithm to form a larger SV, and the rough breakpoints are generated. Since the likelihood ratio test follows the chi-square distribution, SENSV can calculate a *p*-value for the merged SV and consider regions with a *p*-value ≤ 0.05 as SV candidates (in addition to the candidates detected by the split-read alignment). Note that this method can also detect homozygous SV because the distribution for an interval with a homozygous SV is closer to N(*μ*_SV_, *σ*^2^) than N(*μ*, *σ*^2^).

### SV-aware aligner and breakpoint refinement

Many general-purpose aligners, such as Minimap2, calculate the alignment score based on a substitution matrix and gap-scoring scheme. A long deletion is penalized by a large gap extension penalty. This penalty is not desirable in finding the breakpoints of SV. Therefore, we propose an SV-aware aligner, called SV-DP, to find the breakpoints of large SVs.

SV-DP adopts a scoring scheme that does not penalize at most one gapped alignment introduced by an SV. By not penalizing the gapped alignment caused by SV (including SVs other than deletion), the other parts of the read have a higher chance of being aligned correctly to the reference, thus obtaining more precise breakpoints.

The implementation of SV-DP is non-trivial because the time for aligning the read then depends on the size of the largest gap (i.e., the targeted one), which can be as big as several Mbp. To speed up SENSV (while retaining the same level of sensitivity), we perform SV-DP only on sequences near candidate breakpoints. Let *R* be a reference sequence, and let *Q* be a read sequence. Let *w* and *W* be predefined window sizes (2 kbp and 10 kbp, respectively). Assume *Q*[1..*q*] is aligned to *R*[*i*..*s*], and *Q*[*q* + 1..*end*] to *R*[*e*..*j*] in the initial alignment of an SV candidate. We consider *Q*[*q* − *w*.. *q* + *w*] as a query and stitch two pieces of reference *R*[*s* − *w* .. *s* + *W*], *R*[*e* − *W*..*e* + *w*] together as the new reference. In this way, the running time of SV-DP is O(*wW*), which does not depend on SV size. We can recover the exact breakpoint, even if the candidate breakpoint is offset by *w* in the query and/or *W* in the reference.

For candidates found by sequencing depth, where the breakpoint is not accurate, SENSV reuses the seeding results from Minimap2 to find the supporting reads and narrows down the range of breakpoints. We extracted seeding information from Minimap2 in the format of [*query start*, *query end*, *ref start*, *ref end*]. For a candidate from depth analysis, if we can find one read with one seed near the starting breakpoint and another seed near the ending breakpoint, we proceed with this read to SV-DP to refine the breakpoint.

Finally, after all breakpoints are refined, de-duplication is applied to remove duplicate candidates.

### SV validation

Once SENSV has figured out the breakpoints, the SV candidates can be validated using an alternative reference sequence. For an SV candidate, an alternative reference sequence is constructed by extracting nearby sequences from the reference genome and inserting the candidate into the sequence. If there are multiple SV candidates with similar breakpoints, multiple alternative reference sequences are constructed. If the SV candidate is genuine, we expect to see more reads previously aligned to the reference sequence aligned to the alternative sequence. Therefore, a candidate is discarded if its alternative reference fails to attract more reads. To minimize possible errors due to the existence of other SVs, the final set of SV candidates are validated again using an alternative reference sequence and the entire human reference genome. If reads can be confidently aligned to the alternative reference rather than elsewhere in the human reference genome, the corresponding candidate is likely genuine.

Finally, the QUAL score of the SV is derived from the alignment results in the validation process. Features like alignment length in query and reference and the number of matching bases are included to filter false positive SVs.

### Evaluation method

NanoVar was evaluated using the default parameters, with raw reads as input (NanoVar has an internal aligner). For Sniffles and SVIM, as recommended in their paper, we first performed the alignment using NGMLR^10^ and then used the generated bam file as inputs. For cuteSV, we also used NGMLR alignment results as it was benchmarked in its research. Since the default parameters of the two software programs were not optimized for low-dept, to increase sensitivity, we consulted the developers of the software and tried multiple combinations of parameters. The best combination we found for the two software is shown in Supplementary Table S9. For SENSV, the initial alignment was done by Minimap2, and realignment using the SV-DP module was always performed.

The number of SVs reported by each software is calculated by using “Bcftools filter”^26^ to include only the predicted SVs with certain sizes (at least 1000 bp) and SV types, including “DEL”, “DUP”, “INV”, and “BND”. Then, SURVIVOR was primarily used to assess the number of true positives (TP) and the number of false negatives (FN), and the number of false positives (FP) of the filtered results in detecting the SVs. The command “SURVIVOR eval” was used with the allowance of 100-bp or 2000-bp error distance from the breakpoints of the true-sets. This means only the predicted SVs with correct SV type prediction and the breakpoints being within a 100-bp error are considered the true positives (TP). To maximize the sensitivity for translocation, we also considered the predicted SVs, which were classified as “BND”. The example commands used for SURVIVOR and bcftools can be found in Supplementary Table S10.

### Ethical statement

All experimental protocols were reviewed and approved by the University of Hong Kong Human Research Ethics Committee (HREC reference number: EA210242). All methods were performed under relevant guidelines and regulations. Informed consent was not included because the data was unidentifiable and anonymous and was used for bioinformatics method development. The outcomes of the development will not affect the standard of care and management of the current patients.

## Conclusion

SENSV, by integrating several efficient algorithmic techniques, including SV-aware alignment (SV-DP), analysis of sequencing depth information, and sophisticated verification via realignment, can effectively utilize 4× ONT whole genome sequencing data to detect structural variations (size starting from thousands of bp) with superior sensitivity, precision and breakpoint resolution. This makes clinical diagnosis of pathogenic SV using a single MinION flowcell feasible and cost-effective.

## Supplementary Information

Supplementary Information.

## Data Availability

The source code of SENSV is available on Github: github.com/HKU-BAL/SENSV, implemented in Python 3.

## References

[1] Sudmant PH (2015). An integrated map of structural variation in 2,504 human genomes. Nature.

[2] Lappalainen I (2013). DbVar and DGVa: public archives for genomic structural variation. Nucleic Acids Res..

[3] Layer RM, Chiang C, Quinlan AR, Hall IM (2014). LUMPY: a probabilistic framework for structural variant discovery. Genome Biol..

[4] Rausch T (2012). DELLY: structural variant discovery by integrated paired-end and split-read analysis. Bioinformatics.

[5] Chong Z (2017). novoBreak: local assembly for breakpoint detection in cancer genomes. Nat. Methods.

[6] Chen K (2009). BreakDancer: an algorithm for high-resolution mapping of genomic structural variation. Nat. Methods.

[7] Rodriguez OL, Ritz A, Sharp AJ, Bashir A (2020). MsPAC: a tool for haplotype-phased structural variant detection. Bioinformatics.

[8] Kallioniemi A, Visakorpi T, Karhu R, Pinkel D, Kallioniemi O-P (1996). Gene copy number analysis by fluorescencein situhybridization and comparative genomic hybridization. Methods.

[9] Xiao T, Zhou W (2020). The third generation sequencing: the advanced approach to genetic diseases. Transl. Pediatr..

[10] Sedlazeck FJ (2018). Accurate detection of complex structural variations using single-molecule sequencing. Nat. Methods.

[11] Li H (2018). Minimap2: pairwise alignment for nucleotide sequences. Bioinformatics.

[12] Heller D, Vingron M (2019). SVIM: structural variant identification using mapped long reads. Bioinformatics.

[13] Jiang T (2020). Long-read-based human genomic structural variation detection with cuteSV. Genome Biol..

[14] Tham CY (2020). NanoVar: accurate characterization of patients' genomic structural variants using low-depth nanopore sequencing. Genome Biol..

[15] Feuk L, Marshall CR, Wintle RF, Scherer SW (2006). Structural variants: changing the landscape of chromosomes and design of disease studies. Hum. Mol. Genet..

[16] Freeman JL (2006). Copy number variation: new insights in genome diversity. Genome Res..

[17] Mahmoud M (2019). Structural variant calling: the long and the short of it. Genome Biol..

[18] Jeffares DC (2017). Transient structural variations have strong effects on quantitative traits and reproductive isolation in fission yeast. Nat. Commun..

[19] Wang B (2017). Analysis of meiotic segregation patterns and interchromosomal effects in sperm from 13 robertsonian translocations. Balkan J. Med. Genet..

[20] Zook JM (2020). A robust benchmark for detection of germline large deletions and insertions. Nat. Biotechnol..

[21] Bartenhagen C, Dugas M (2013). RSVSim: an R/Bioconductor package for the simulation of structural variations. Bioinformatics.

[22] Yang C, Chu J, Warren RL, Birol I (2017). NanoSim: nanopore sequence read simulator based on statistical characterization. Gigascience.

[23] Jain M (2018). Nanopore sequencing and assembly of a human genome with ultra-long reads. Nat. Biotechnol..

[24] Krusche P (2019). Best practices for benchmarking germline small-variant calls in human genomes. Nat. Biotechnol..

[25] Li H (2016). Minimap and miniasm: fast mapping and de novo assembly for noisy long sequences. Bioinformatics.

[26] Li H (2009). The Sequence Alignment/Map format and SAMtools. Bioinformatics.

