# Supplementary material for: Detecting structural variations with precise breakpoints using low-depth WGS data from a single oxford nanopore MinION flowcell
Source: Sci Rep. 2022 Mar 16;12:4519. doi: 10.1038/s41598-022-08576-4 (PMC8927474; doi:10.1038/s41598-022-08576-4)
Supplement: Supplementary file 1 — Supplementary Information. [file 41598_2022_8576_MOESM1_ESM.pdf]

Supplementary Material

Detecting Structural Variations with Precise Breakpoints using Low-Depth WGS Data from a Single Oxford Nanopore MinION Flowcell

Henry CM Leung[1,†], Huijing Yu[1,†], Yifan Zhang[1,†], Wing Sze Leung[1], Ivan FM Lo[2], Ho Ming Luk[2], Wai-Chun Law[3], Ka Kui Ma[1], Chak Lim Wong[1], Yat Sing Wong[1], Ruibang Luo[1,*], Tak-Wah Lam[1,*]

[1]Department of Computer Science, The University of Hong Kong, Hong Kong

[2]Clinical Genetic Service, Department of Health, Hong Kong

[3]L3 Bioinformatics Limited, Hong Kong

*Corresponding author contact: rbluo@cs.hku.hk

*Corresponding author contact: twlam@cs.hku.hk

[†]these authors contributed equally to this work

Table of Contents

S.1. The number of reported SV in different types detected by the software for the datasets 3

S.2. Performance of the software using simulated datasets and HG002 8

S.3. Performance of SENSV, NanoVar[1], Sniffles[2], SVIM[3] and cuteSV[4] on the simulated datasets for individual SV 9

S.4. CPU Usage of SENSV for Simulated Datasets and low-depth HG002 11

S.5. The Algorithm of SV-DP 12

S.6. Depth variability of the reference dataset in SENSV 14

S.7.Parameters and software version used for calling SV for low-depth (4x) WGS data 15

S.8. Commands and parameters used for the evaluation 16

Reference 17

S.1. The number of reported SV in different types detected by the software for the datasets

	Number of predicted deletions				
Dataset ID	SENSV	NanoVar	Sniffles	SVIM	cuteSV
HG002	1,850	1,209	984	2,280	843
simulated data A	81	37	52	200	29
simulated data B	55	26	46	212	22
1	1,513	1,278	1,027	2,078	831
2	1,431	1,353	1,172	2,129	945
3	1,743	1,146	732	2,207	599
4	1,612	1,176	794	2,036	658
5	3,395	1,514	1,062	4,970	848
6	17,209	3,692	1,375	17,783	834
7	1,353	1,192	973	1,715	791
8	1,792	1,320	1,192	2,983	1,016
9	1,515	1,294	1,014	1,982	786
10	2,868	1,474	1,362	4,534	1,090
11	1,675	1,282	805	2,489	640
12	2,195	1,428	779	2,832	639
13	3,476	1,498	1,510	4,649	1,168
14	35,848	4,137	2,118	35,169	950
15	1,413	1,220	968	2,031	798
16	2,275	1,355	1,301	3,683	1,059
17	3,930	1,437	1,657	6,666	1,342
18	1,750	1,404	1,294	2,781	1,091
19	1,100	1,165	455	973	362
20	1,543	1,211	964	1,767	800
21	2,197	1,423	1,359	3,411	1,225
22	1,195	1,183	786	1,425	644
23	2,002	1,317	965	2,085	792
24	2,218	1,366	992	2,670	835

Table S1. The number of reported deletions by each software.

	Number of predicted duplications				
Dataset ID	SENSV	NanoVar	Sniffles	SVIM	cuteSV
HG002	285	1,882	759	2,730	1,837
simulated data A	31	27	36	216	26
simulated data B	29	40	41	242	36
1	190	2,225	784	2,191	2,516
2	209	2,502	903	2,370	2,825
3	140	1,568	514	2,006	1,683
4	190	1,837	572	1,950	1,964
5	276	3,017	776	3,501	1,839
6	218	2,962	755	2,042	2,094
7	200	1,779	667	1,901	2,105
8	200	2,236	933	3,120	2,473
9	160	2,562	697	1,914	1,823
10	276	3,030	1,085	3,805	2,966
11	139	2,128	599	2,075	1,833
12	137	2,245	621	2,195	2,081
13	333	4,177	1,246	3,825	3,773
14	283	2,769	843	2,692	2,404
15	154	2,208	704	2,064	1,627
16	236	2,606	972	3,591	2,691
17	506	3,672	1,145	6,787	3,520
18	182	2,662	872	3,159	2,552
19	149	1,610	247	928	1,040
20	175	2,062	610	1,765	1,738
21	246	2,670	935	4,037	2,735
22	155	1,756	535	1,556	1,353
23	157	2,244	665	1,838	2,126
24	240	2,572	679	2,240	2,108

Table S2. The number of reported duplications by each software.

	Number of predicted inversions				
Dataset ID	SENSV	NanoVar	Sniffles	SVIM	cuteSV
HG002	233	1,261	634	351	637
simulated data A	11	20	69	115	56
simulated data B	7	24	85	129	61
1	141	1,194	600	439	608
2	72	1,360	708	463	722
3	103	975	478	428	466
4	159	1,076	448	394	484
5	167	1,280	700	502	675
6	256	1,256	594	420	596
7	96	1,071	573	329	604
8	226	1,194	776	574	822
9	176	1,240	597	404	625
10	411	1,381	861	645	922
11	219	1,153	444	324	496
12	125	1,236	476	370	496
13	271	1,615	986	697	1,017
14	233	1,244	724	579	767
15	240	1,163	636	453	636
16	167	1,286	897	660	937
17	114	1,413	1,391	849	1,336
18	118	1,333	853	523	903
19	192	1,258	190	117	210
20	207	1,112	566	346	606
21	160	1,437	1,026	633	1102
22	98	1,052	444	255	482
23	177	1,275	545	303	582
24	237	1,278	589	329	577

Table S3. The number of reported inversions by each software.

	Number of predicted translocations (reported as "BND")				
Dataset ID	SENSV	NanoVar	Sniffles	SVIM	cuteSV
HG002	1,276	8,422	1,609	1,289	1,550
simulated data A	21	150	61	14	30
simulated data B	20	170	65	12	34
1	116	5,720	1,543	1,363	1,488
2	101	6,238	1,718	1,581	1,677
3	209	5,942	1,185	941	1,004
4	91	5,254	1,206	1,110	1,122
5	1,101	8,832	2,035	1,690	1,876
6	164	5,938	1,547	1,284	1,421
7	92	5,230	1,375	1,076	1,259
8	867	6,998	2,138	1,917	2,062
9	80	5,672	1,501	1,334	1,443
10	751	6,970	2,265	1,964	2,250
11	490	7,106	1,383	1,154	1,310
12	462	7,802	1,432	1,179	1,326
13	682	7,058	2,486	2,085	2,404
14	269	6,156	1,719	1,429	1,595
15	128	5,512	1,401	1,182	1,314
16	883	6,688	2,340	2,052	2,263
17	972	7,378	3,358	2,505	3,422
18	1,053	6,976	2,451	1,826	2,336
19	87	4,904	655	382	576
20	89	5,554	1,463	1,190	1,404
21	861	6,898	2,594	2,012	2,518
22	76	5,254	1,187	996	1,084
23	111	5,542	1,449	1,065	1,319
24	592	6,628	1,775	1,358	1,645

Table S4. The number of reported translocations by each software.

	Total				
Dataset ID	SENSV	NanoVar	Sniffles	SVIM	cuteSV
HG002	3,644	12,774	3,986	6,650	4,867
simulated data A	144	234	218	545	141
simulated data B	111	260	237	595	153
1	1,960	10,417	3,954	6,071	5,443
2	1,813	11,453	4,501	6,543	6,169
3	2,195	9,631	2,909	5,582	3,752
4	2,052	9,343	3,020	5,490	4,228
5	4,939	14,643	4,573	10,663	5,238
6	17,847	13,848	4,271	21,529	4,945
7	1,741	9,272	3,588	5,021	4,759
8	3,085	11,748	5,039	8,594	6,373
9	1,931	10,768	3,809	5,634	4,677
10	4,306	12,855	5,573	10,948	7,228
11	2,523	11,669	3,231	6,042	4,279
12	2,919	12,711	3,308	6,576	4,542
13	4,762	14,348	6,228	11,256	8,362
14	36,633	14,306	5,404	39,869	5,716
15	1,935	10,103	3,709	5,730	4,375
16	3,561	11,935	5,510	9,986	6,950
17	5,522	13,900	7,551	16,807	9,620
18	3,103	12,375	5,470	8,289	6,882
19	1,528	8,937	1,547	2,400	2,188
20	2,014	9,939	3,603	5,068	4,548
21	3,464	12,428	5,914	10,093	7,580
22	1,524	9,245	2,952	4,232	3,563
23	2,447	10,378	3,624	5,291	4,819
24	3,287	11,844	4,035	6,597	5,165

Table S5. The number of total reported SV by each software.

S.2. Performance of the software using simulated datasets and HG002

Datasets	Software	F1%		Precision%		Recall%	
		100bp	2000bp	100bp	2000bp	100bp	2000bp
Simulated Dataset A	SENSV	42.29%	42.49%	27.78%	27.78%	88.57%	88.57%
	NanoVar	17.25%	17.25%	10.26%	10.26%	54.29%	54.29%
	Sniffles	14.23%	15.02%	8.26%	8.72%	51.43%	54.29%
	SVIM	6.79%	7.45%	3.67%	4.04%	45.71%	48.57%
	cuteSV	28.21%	28.21%	18.44%	18.44%	60.00%	60.00%
Simulated Dataset B	SENSV	49.12%	49.12%	36.04%	36.04%	77.14%	77.14%
	NanoVar	12.36%	12.36%	7.31%	7.31%	40.00%	40.00%
	Sniffles	11.03%	11.03%	6.33%	6.33%	42.86%	42.86%
	SVIM	6.55%	6.55%	3.35%	3.53%	45.71%	45.71%
	cuteSV	15.51%	15.51%	9.80%	9.80%	37.14%	37.14%
HG002	SENSV	32.75%	37.75%	21.08%	24.59%	73.31%	81.20%
	NanoVar	41.11%	46.71%	29.78%	34.66%	66.35%	71.62%
	Sniffles	35.49%	41.29%	27.34%	31.81%	50.56%	58.83%
	SVIM	26.96%	31.58%	16.62%	19.65%	71.24%	80.45%
	cuteSV	39.56%	43.12%	32.27%	35.35%	51.13%	55.26%

Table S6. The F1%, precision% and recall% for all software when using simulated datasets and HG002.

S.3. Performance of SENSV, NanoVar[1], Sniffles[2], SVIM[3] and cuteSV[4] on the simulated datasets for individual SV

SV ID	SV length	SV type	SENSV (heterozygous datasets)	SENSV (homozygous datasets)	NanoVar	Sniffles	SVIM	cuteSV
1	39,438	deletion	Y	Y	Y	Y	Y	Y
2	82,549	deletion	Y	Y	N	N	Y	N
3	94,159	deletion	Y	Y	Y	Y	Y	Y
4	10,117	deletion	Y	Y	Y	Y	Y	Y
5	48,202	deletion	Y	Y	N	Y	Y	Y
6	33,546	deletion	Y	Y	N	N	Y	N
7	23,876	deletion	Y	Y	N	N	Y	N
8	97,991	deletion	Y	Y	Y	Y	Y	Y
9	34,940	deletion	Y	Y	N	Y	Y	Y
10	62,420	deletion	Y	Y	N	Y	Y	N
11	113,720	long deletion	Y	Y	Y	Y	Y	Y
12	838,659	long deletion	Y	Y	Y	Y-	Y-	N
13	498,160	long deletion	Y	Y	N	Y	N	Y
14	1,095,657	long deletion	Y	Y	N	N	Y	N
15	412,281	long deletion	Y	Y	Y	N	N	N
16	937,186	long deletion	Y	Y	Y	Y	Y	Y
17	509,473	long deletion	Y	Y	N	N	N	N
18	1,063,340	long deletion	N	N	N	N	N	N
19	119,059	long deletion	Y	Y	Y	N	Y	N
20	1,045,185	long deletion	Y	Y	Y	N	Y	N
21	940,447	inversion	Y	Y	Y	Y	N	Y
22	930,177	inversion	Y	Y	Y	Y	Y	Y
23	985,020	inversion	Y	Y	Y	Y	Y	Y
24	43,362	inversion	Y	Y	Y	Y	Y	Y
25	43,233	inversion	Y	Y	Y	Y	Y	Y
26	40,819	inversion	N	Y	N	N	N	N
27	45,234	inversion	Y	Y	Y	Y	N	Y
28	41,191	inversion	Y	Y	Y	Y	N	N
29	909,789	inversion	Y	Y	Y	Y	Y	Y
30	940,949	inversion	Y	Y	Y	Y	N	Y
31	470,496	duplication	Y	Y	N	N	N	N
32	80,807	duplication	Y	Y	Y	Y	Y	Y
33	116,822	duplication	Y	Y	N	Y	Y	Y
34	47,269	duplication	Y	Y	Y	Y	Y	Y
35	56,779	duplication	Y	Y	Y	N	N	N

SV ID	SV length	SV type	SENSV (heterozygous datasets)	SENSV (homozygous datasets)	NanoVar	Sniffles	SVIM	cuteSV
36	896,748	duplication	Y	Y	N	N	Y	N
37	1,189,698	duplication	Y	Y	N	Y	Y	Y
38	55,797	duplication	N	Y	N	N	N	N
39	499,008	duplication	N	Y	N	N	N	N
40	40,566	duplication	Y	Y	Y	N	N	N
41	850,000	terminal deletion	Y	Y	N	N	N	N
42	600,000	terminal deletion	Y	Y	N	N	N	Y
43	1,000,000	terminal deletion	Y	Y	N	N	N	Y
44	800,000	terminal deletion	N	Y	N	N	N	Y
45	900,000	terminal deletion	Y	Y	N	N	N	N
46	2,500,000	terminal deletion	N	N	N	N	N	N
47	3,000,000	terminal deletion	Y	Y	N	N	Y	N
48	1,500,000	terminal deletion	Y	Y	N	N	Y	N
49	1,000,000	terminal deletion	Y	Y	N	N	N	N
50	2,720,000	terminal deletion	N	Y	N	N	N	N
51	N/A	balanced translocation	Y	Y	Y	Y	Y	Y
52	N/A	balanced translocation	N	Y	N	N	N	N
53	N/A	balanced translocation	Y	Y	Y	Y	N	Y
54	N/A	balanced translocation	Y	Y	Y	Y	N	Y
55	N/A	balanced translocation	N	Y	Y	N	N	N
56	N/A	balanced translocation	Y	Y	Y	Y	N	Y
57	N/A	balanced translocation	Y	Y	Y	Y	N	Y
58	N/A	balanced translocation	Y	Y	N	N	N	N
59	N/A	balanced translocation	Y	Y	Y	Y	Y	Y
60	N/A	balanced translocation	Y	Y	Y	Y	Y	Y
61	N/A	unbalanced translocation	Y	Y	N	N	N	N
62	N/A	unbalanced translocation	Y	Y	Y	Y	Y	Y
63	N/A	unbalanced translocation	Y	Y	N	N	N	N
64	N/A	unbalanced translocation	Y	Y	N	N	N	N
65	N/A	unbalanced translocation	N	Y	N	N	N	N
66	N/A	unbalanced translocation	Y	Y	Y	Y	Y	Y
67	N/A	unbalanced translocation	N	Y	N	N	N	N
68	N/A	unbalanced translocation	Y	Y	N	N	N	N
69	N/A	unbalanced translocation	N	N	N	N	N	N
70	N/A	unbalanced translocation	Y	Y	N	Y	N	Y

Table S7. Comparison of SENSV, NanoVar, Sniffles and SVIM on the ability to detect the SV in the simulated datasets. Above, "Y" [and "Y-"] mean that a software can detect the SV with the correct SV type and with a breakpoint precision of 100 bp [and of 2,000 bp]; and "N" indicates the software is unable to detect the SV with breakpoint precision relaxed to 2,000 bp. Simulated datasets with SVs that share same breakpoints as before but homozygous are run with SENSV to show SENSV's ability to detect homozygous SVs.

S.4. CPU Usage of SENSV for Simulated Datasets and low-depth HG002

	Dataset	CPU usage
SENSV	Simulated Dataset A	3,451.22%
	Simulated Dataset B	2,381.96%
	HG002	2,972.94%

Table S8. The CPU usage of SENSV for simulated datasets and HG002.

S.5. The Algorithm of SV-DP

Let Q = q1q2 ... qn and R = r1r2...rn be query sequence and reference sequence respectively, where n and m are the lengths of the sequences.

Substitution matrix s(q,r) defines the similarity score of two nucleotides q and r,
and penalty function W(k) defines the penalty of a gap (deletion or insertion) with length k.

Scoring matrix H of our dynamic programming is a 3 dimensional table, and consists of 4 subtables.

H[k][i][j] represents the best alignment of Q[1..i] and R[1..j], with exactly k of the deletions has a zero penalty. In our SV detection application, only 1 free deletion is needed, and k will be either 0 or 1. In implementation, we use a circular buffer of size 2 (instead of size = query length) to represent the second dimension.

H_M[k][i][j] stores the best alignment that ends in match/mismatch (i.e. Q[i] is aligned to R[j])

H_I[k][i][j] stores the best alignment that ends in insertion (i.e. Q[i] is in an insertion), while insertion length is recorded in a separate table I_len[k][j][i].

H_D[k][i][j] stores the best alignment that ends in deletion (i.e. R[j] is in a deletion), while deletion length is recorded in a separate table D_len[k][j][k]

H_F[k][i][j] stores the best alignment that ends in deletion and the deletion is of free penalty.

For all these 4 subtables, we stores not only scores, but also the positions of the deletion with free penalty, if any (starting position in reference, ending position in reference, query position).

We keep track of the best alignment while filling the scoring matrices and therefore a backtracking would not be necessary to retrieve the deletion with free penalty.

Initialization:

```
H_M[k][j][i] = 0,
H_I[k][j][i] = -inf,
H_D[k][j][i] = -inf,
```

```
H_F[k][j][i] = -inf,

I_len[k][j][i] = D_len[k][j][i] = -inf


Transitions:

for i>1, j>1

(H_M is written as M)

I[k][i][j] = max(M[k][i-1][j] + W(1), I[k][i-1][j] + W(l+1) -
W(l)) where l = I_len[k][i-1][j]
D[k][i][j] = max(M[k][i][j-1] + W(1), D[k][i][j-1] + W(l+1) -
W(l)) where l = D_len[k][i][j-1]
if k>0:
    F[k][i][j] = max(M[k-1][i][j-1], F[k-1][i][j-1])
M[k][i][j] = max(I[k][i][j], D[k][i][j], F[k][i][j], M[k][i-
1][j-1] + s(Q[i],R[j]))

(supplementary information like I_len, D_len, and position of
deletion of free penalty are updated accordingly.)

RET = max(RET, M[k][i][j])


Return Value:
RET
```

S.6. Depth variability of the reference dataset in SENSV

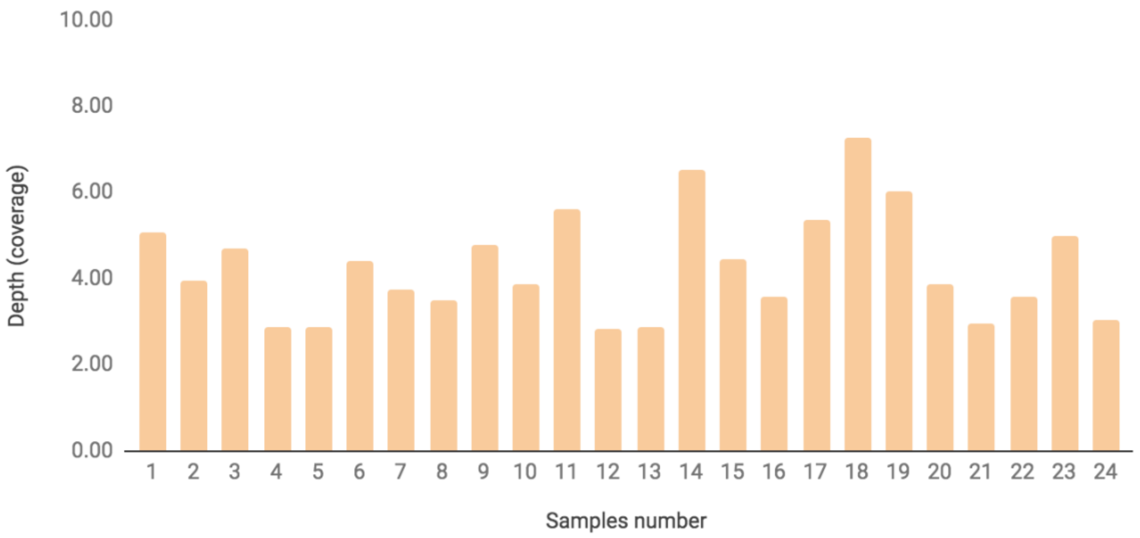

Figure S1. Sequencing depth of the samples for the reference dataset in SENSV

S.7.Parameters and software version used for calling SV for low-depth (4x) WGS data

Software	Parameters
SENSV	--min_sv_size 1000
Sniffles (1.0.11)	-s 2
SVIM (1.1.1)	alignment --max_sv_size 250000000
NanoVar (1.2.6)	-r hs37d5.fa
ngmlr (0.2.7)	-x ont
Minimap2 (2.17)	-Y -t ${thread} -z 200 --MD -a -5
cuteSV (1.0.11)	--max_cluster_bias_INS 100 –diff_ratio_merging_INS 0.3 –max_cluster_bias_DEL 100 –diff_ratio_merging_DEL 0.3 –min_support 2 –max_sv_size 250000000

Table S9. Parameters and versions used for the software when evaluating low-depth WGS data.

S.8. Commands and parameters used for the evaluation

Software	Command
SURVIVOR[5] (1.0.7)	SURVIVOR eval --vcf_results --true_set_bed2_format --100 (allowed error at the breakpoints) --output_dir
bcftools[6]	bcftools filter --i 'SVTYPE==(Type)'--vcf | bcftools filter --i 'SVLEN>=1000' or 'SVLEN<=-1000' (for deletions)

Table S10. Commands and parameters used for counting the number of reported SV and true positives. The bcftools command has been used to gather the number of reported SV in different types for Section S.1 (Table S1 to Table S4).

Reference

1 Tham, C. Y. *et al.* NanoVar: accurate characterization of patients' genomic structural variants using low-depth nanopore sequencing. *Genome Biol* **21**, 56, doi:10.1186/s13059-020-01968-7 (2020).

2 Sedlazeck, F. J. *et al.* Accurate detection of complex structural variations using single-molecule sequencing. *Nat Methods* **15**, 461-468, doi:10.1038/s41592-018-0001-7 (2018).

3 Heller, D. & Vingron, M. SVIM: structural variant identification using mapped long reads. *Bioinformatics* **35**, 2907-2915, doi:10.1093/bioinformatics/btz041 (2019).

4 Jiang, T. *et al.* Long-read-based human genomic structural variation detection with cuteSV. *Genome Biol* **21**, 189, doi:10.1186/s13059-020-02107-y (2020).

5 Jeffares, D. C. *et al.* Transient structural variations have strong effects on quantitative traits and reproductive isolation in fission yeast. *Nat Commun* **8**, 14061, doi:10.1038/ncomms14061 (2017).

6 Li, H. *et al.* The Sequence Alignment/Map format and SAMtools. *Bioinformatics* **25**, 2078-2079, doi:10.1093/bioinformatics/btp352 (2009).
